# Evaluation of Respiratory Syncytial Virus Prefusion F IgG Antibodies in Japanese Mother–Infant Pairs Following Maternal Abrysvo Vaccination and in Infants Hospitalized with RSV Infection

**DOI:** 10.3390/vaccines14090780

**Published:** 2026-09-06

**Authors:** Tomohiro Oishi, Takashi Nakano

**Affiliations:** 1 Department of Clinical Infectious Diseases, Kawasaki Medical School, 577, Matsushima, Kurashiki 701-0192, Okayama, Japan; 2 Department of Pediatrics, Kawasaki Medical School, 577, Matsushima, Kurashiki 701-0192, Okayama, Japan; nakano@med.kawasaki-m.ac.jp

**Keywords:** respiratory syncytial virus, maternal immunization, Abrysvo, transplacental antibody transfer, post-COVID-19, pediatrics

## Abstract

**Background/Objectives**: The approval of Abrysvo, a maternal bivalent respiratory syncytial virus (RSV) prefusion F (pre-F) vaccine, marked a major milestone in pediatric prophylaxis. However, real-world serological data on transplacental antibody transfer in Asian populations remains limited. This brief report evaluated serum RSV pre-F immunoglobulin G (IgG) geometric mean titers (GMTs) across three distinct Japanese cohorts. **Methods**: This single-center, comparative study analyzed neonates born to Abrysvo-vaccinated mothers (*n* = 12), neonates born to unvaccinated mothers (*n* = 40), and infants aged <6 months hospitalized with laboratory-confirmed RSV infection (*n* = 18) using enzyme-linked immunosorbent assay (ELISA). **Results**: Neonates of vaccinated mothers exhibited significantly higher pre-F IgG GMTs (4.74; 95% CI: 4.30–5.18) than those of unvaccinated mothers (3.80; 95% CI: 3.50–4.14). Conversely, infants requiring hospitalization for RSV infection demonstrated critically low pre-F IgG GMTs (1.08; 95% CI: 0.65–2.15), revealing statistically significant differences across all three groups (*p* < 0.001). **Conclusions**: Maternal immunization with Abrysvo significantly enhances passive humoral immunity in Japanese neonates compared to natural background immunity. Given the severe antibody deficiency observed in hospitalized infants, expanding maternal vaccination coverage is a high-priority public health strategy to mitigate the pediatric RSV burden in the post-pandemic era.

## 1. Introduction

Respiratory syncytial virus (RSV) is a leading cause of acute lower respiratory tract infections (ALRTIs) in infants and young children worldwide and is associated with substantial morbidity, hospitalization, and mortality [1,2]. The risk of severe RSV infection, including bronchiolitis and pneumonia, is highest during the first six months of life, when the infant’s immune system is immature and largely dependent on maternally transferred antibodies.

The epidemiological landscape of RSV has shifted dramatically since the coronavirus disease 2019 (COVID-19) pandemic. Non-pharmaceutical interventions (NPIs) implemented to curb SARS-CoV-2 transmission suppressed seasonal RSV circulation, leading to an expanded cohort of immunologically naive pregnant women and young children [3]. Consequently, the post-pandemic period has been characterized by typical, severe, and out-of-season RSV resurgences globally and in Japan, underscoring the urgent need for enhanced protective strategies for vulnerable neonates [4].

Historically, passive immunoprophylaxis was limited to high-risk infants receiving palivizumab and, more recently, long-acting monoclonal antibodies such as nirsevimab. However, universal protection remained an unfulfilled goal until the development and approval of maternal RSV vaccines. Abrysvo, a bivalent RSV prefusion F protein vaccine, was approved in Japan in 2024 for maternal immunization during late pregnancy to protect infants from birth through six months of age via transplacental antibody transfer. While clinical trials have demonstrated high efficacy, real-world serological data evaluating RSV prefusion F (pre-F) immunoglobulin G (IgG) titers in Japanese mother–infant pairs remain limited, particularly studies directly comparing vaccinated and unvaccinated cohorts and infants hospitalized with severe RSV infection in the post-pandemic era [1,5,6].

Therefore, this study aimed to evaluate and compare serum RSV pre-F IgG geometric mean titers (GMTs) among three distinct groups at a single academic center in Japan: neonates born to Abrysvo-vaccinated mothers, neonates born to unvaccinated mothers, and infants younger than six months hospitalized with RSV infection. This study provides important clinical insights into the immunogenicity of maternal vaccination and the humoral protection associated with hospitalization due to severe RSV infection.

## 2. Materials and Methods

### 2.1. Ethical Considerations

Informed consent was obtained from the parents or legal guardians of all participants. The study was approved by the Ethics Committee of Kawasaki Medical School, Kurashiki, Japan, on 10 March 2026 (approval no. 5370-07).

### 2.2. Study Population and Cohorts

Neonates whose mothers were admitted for delivery at the Kawasaki Medical School Hospital were enrolled. Among them, neonates born to mothers who were not vaccinated with Abrysvo were enrolled from March 2023 to February 2024, whereas those born to mothers vaccinated with Abrysvo were enrolled from August 2024 to March 2026. Furthermore, infants younger than six months who were admitted with RSV infection between August 2022 and November 2024 were enrolled. RSV infection was laboratory-confirmed in all hospitalized infants using a high-specificity rapid antigen detection test on nasopharyngeal swab specimens collected at admission. Severe RSV-associated lower respiratory tract illness (LRTI) was defined based on clinical criteria requiring hospitalization for severe bronchiolitis or pneumonia presenting with significant respiratory distress, including tachypnea, chest wall retractions, or hypoxia (SpO_2_ < 92% on room air) necessitating oxygen therapy, consistent with previous protocols [1].

Blood and cord samples were obtained from pregnant women at delivery, and blood samples were obtained from pediatric inpatients with RSV infection at admission.

Gestational age at delivery, Apgar score at 5 min after birth, birth weight, outcome, use of palivizumab or nirsevimab, and the presence of siblings were analyzed. In addition, the sex, age, type of delivery, underlying disease, presence of siblings, severity of RSV infection, and use of immunoprophylaxis (palivizumab or nirsevimab) were checked for the hospitalized infant cohort, emphasizing that this infected cohort was entirely distinct from the healthy neonatal cohorts evaluated at birth.

### 2.3. Measurement of IgG Antibody by ELISA on Respiratory Syncytial Virus (RSV)

Serum IgG titers against the RSV pre-fusion F0 protein were determined using the Human Anti-RSV-F0 Antibody IgG Titer ELISA Kit (ACROBiosystems, Newark, DE, USA, Cat. No. RAS-T175), according to the manufacturer’s instructions, as previously described in seroepidemiological studies [7].

### 2.4. Statistical Analysis

GraphPad Prism 5 (GraphPad Software Inc., San Diego, CA, USA) was used for the statistical analysis. Data normality was assessed using the Shapiro–Wilk test. Continuous variables were analyzed using Student’s *t* or Mann–Whitney *U* tests, whereas categorical variables were compared using chi-squared or Fisher’s exact tests. RSV antibody titers were log-transformed to calculate geometric mean titers (GMTs) with 95% confidence intervals (CIs). Differences among the three groups were analyzed using one-way ANOVA followed by Tukey’s post-hoc test for multiple comparisons. Statistical significance was set at *p* < 0.05.

## 3. Results

### 3.1. Participants

A total of 12 neonates born to mothers vaccinated with Abrysvo, 40 neonates born to unvaccinated mothers, and 18 infants with RSV infections were enrolled. All participants were admitted to Kawasaki Medical School Hospital.

### 3.2. Characteristics of Neonates Born to Mothers with or Without Abrysvo Vaccination

The baseline characteristics of healthy neonates at birth are summarized in Table 1. No significant differences were observed between the vaccinated (*n* = 12) and unvaccinated (*n* = 40) groups. Maternal and neonatal parameters were comparable with respect to median gestational age at delivery (38 vs. 38 weeks; *p* = 0.58) and male sex (5/12 [41.7%] vs. 26/40 [65.0%]; *p* = 0.19). The median birth weight was 2916 g in the vaccinated group and 2844 g in the unvaccinated group (*p* = 0.49).

### 3.3. Characteristics of Infants Younger than Six Months Hospitalized with RSV Infection

Table 2 summarizes the characteristics of an independent cohort of 18 infants hospitalized with severe RSV infection within the first six months of life. Among these 18 hospitalized infants, 13 (72.2%) were male. The median (IQR) age at admission was 2 (1–4) months. Crucially, none of these hospitalized infants had a history of receiving immunoprophylaxis with palivizumab or nirsevimab, and none of their mothers had received maternal Abrysvo vaccination during pregnancy, distinguishing this infected cohort from the healthy neonatal cohorts evaluated at birth.

### 3.4. RSV pre-F IgG GMTs in Neonates Born to Vaccinated vs. Unvaccinated Mothers and in Infants Younger than Six Months Hospitalized with RSV Infection

**Figure 1:**
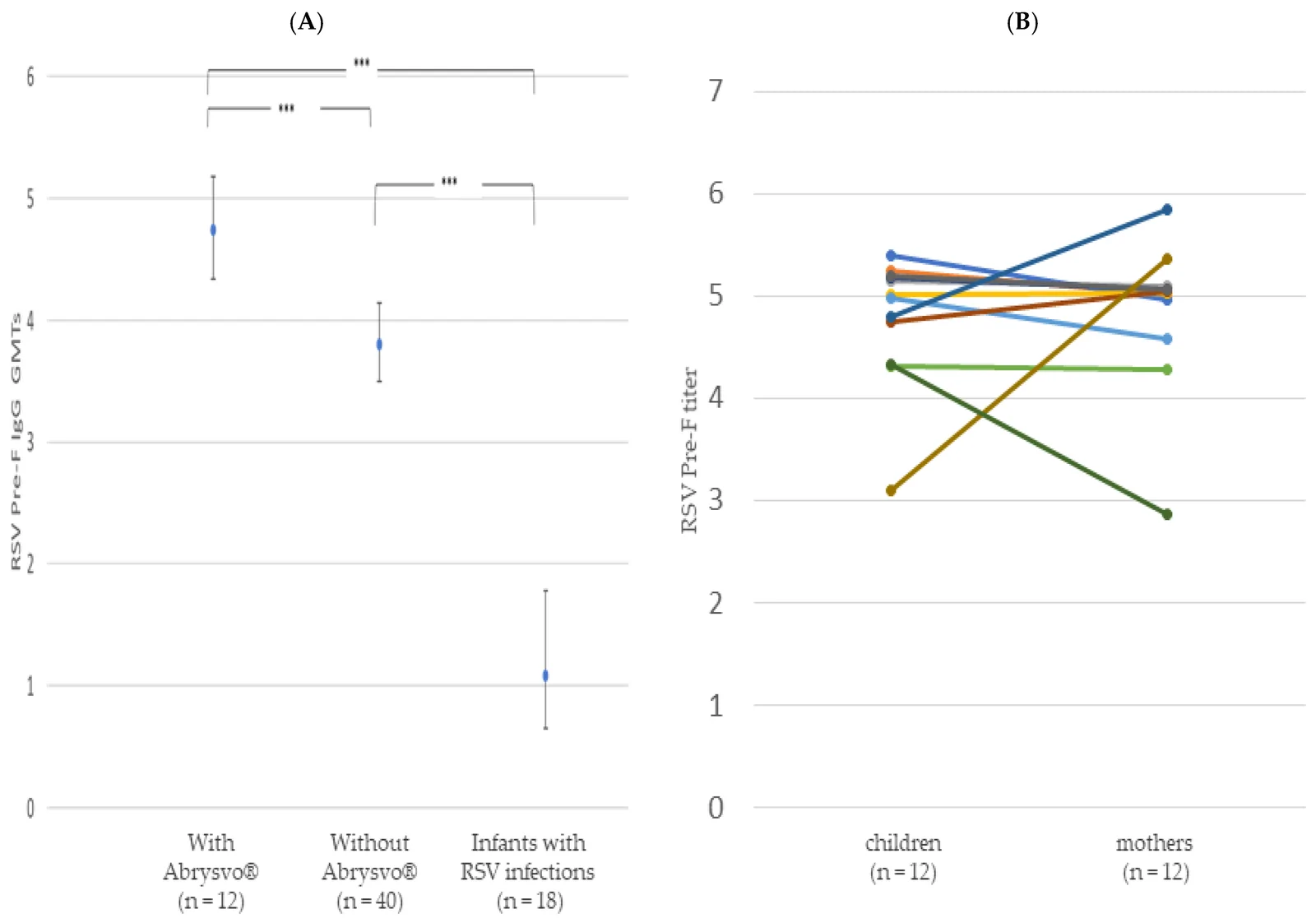
Serological evaluation of serum RSV pre-F IgG levels across cohorts. (**A**) Comparison of geometric mean titers (GMTs) with 95% confidence intervals among neonates born to vaccinated mothers, neonates born to unvaccinated mothers, and infants hospitalized with RSV infection within the first six months of life (*p* < 0.001). (**B**) Paired maternal peripheral blood and neonatal cord blood RSV pre-F IgG titers following maternal Abrysvo vaccination, demonstrating placental antibody transfer trends. ^®^: Registered trademark; *** *p* < 0.001.

RSV pre-F IgG GMTs in neonates born to vaccinated versus unvaccinated mothers and in infants hospitalized with RSV infection within six months of age are shown in Figure 1A. The GMTs (95% CI) in neonates born to vaccinated mothers, neonates born to unvaccinated mothers, and infants hospitalized with RSV infection were 4.74 (4.30–5.18), 3.80 (3.50–4.14), and 1.08 (0.65–2.15), respectively. Significant differences were observed among all three groups (*p* < 0.001).

### 3.5. RSV Prefusion F IgG GMTs in Neonates Born to Mothers with or Without Abrysvo Vaccination and in Infants Younger than Six Months Hospitalized for RSV Infection by Neonatal Characteristics

Table 3 shows RSV pre-F IgG GMTs in neonates born to mothers with or without Abrysvo vaccination and in infants hospitalized with RSV infection within six months of age. RSV pre-F IgG GMTs were analyzed according to gestational age at birth, birth weight, type of delivery, and the presence of siblings. In addition, the interval between Abrysvo vaccination and delivery was analyzed in neonates, and the severity of RSV-associated lower respiratory tract illness was analyzed in infants hospitalized with RSV infection within six months of age. No significant differences were found for any of the background factors.

### 3.6. Comparison of RSV pre-F IgG Titers Between Neonates and Mothers Vaccinated with Abrysvo

Figure 1B shows the comparison of RSV pre-F IgG titers between neonates and mothers vaccinated with Abrysvo. Transplacental transfer rates (the ratio of RSV prefusion F IgG titers of children to that of the mother) were also calculated. Most transfer rates were approximately 1.00 or greater than 1.00, whereas one was 0.58 and another was 1.51.

## 4. Discussion

This study evaluated the serological landscape of RSV protection in Japanese infants during the post-COVID-19 pandemic era by comparing maternal-vaccine-induced antibody levels against natural background immunity with the titers observed in hospitalized infants [1]. Our primary findings revealed that neonates born to mothers vaccinated with Abrysvo had significantly higher RSV pre-F IgG GMTs (4.74; 95% CI: 4.30–5.18) than those born to unvaccinated mothers (3.80; 95% CI: 3.50–4.14). Infants younger than six months who required hospitalization for RSV infection exhibited markedly low pre-F IgG GMTs (1.08; 95% CI: 0.65–2.15). The highly significant differences (*p* < 0.001) among the groups provide strong serological evidence supporting the clinical utility of universal maternal immunization.

The markedly lower antibody titers observed in the hospitalized infant cohort underscore the vulnerability of infants who lack sufficient passive humoral protection. However, it must be noted that while the vaccinated and unvaccinated cohorts were sampled at birth (cord blood), the hospitalized RSV cohort had a median age of 2 months at admission. Because maternally derived IgG naturally decays during the first few months of life, the lower antibody levels observed in the hospitalized group partially reflect this physiological decline and cannot be solely interpreted as a pre-existing antibody deficiency at birth. Nevertheless, our findings demonstrate that natural background immunity often leaves infants with critically low antibody levels (GMT 1.08) by 2 months of age, rendering them highly susceptible to severe community-acquired RSV LRTIs [8,9]. The paired mother–infant data (Figure 1B) demonstrate the efficiency of transplacental antibody transport following Abrysvo vaccination. Maternally synthesized IgG antibodies are actively transported across the placenta into the fetal circulation mediated by the neonatal Fc receptor (FcRn) [1,6,7,10]. This active transport mechanism is concentration-dependent and specific to the IgG subclass, typically resulting in neonatal cord blood titers that equal or exceed maternal circulating levels at delivery. The robust neonatal GMTs (4.74) achieved in the vaccinated group suggest that maternal immunization successfully elevates initial antibody repositories at birth. Consequently, even after accounting for the expected natural decay of IgG over time, infants from vaccinated mothers are anticipated to maintain protective antibody concentrations well above the critical threshold associated with hospitalization throughout their highest-risk window of early infancy. Although monoclonal antibodies such as nirsevimab offer an alternative strategy for infant protection, maternal immunization with Abrysvo offers practical advantages, including immediate protection from birth without the need for infant injection at delivery. Our demographic data showed no statistically significant differences in gestational age, birth weight, or delivery type between the vaccinated and unvaccinated cohorts, suggesting that the GMT disparities were primarily dependent on maternal vaccination status, independent of neonatal profiles [11].

This study has several limitations. First, it was conducted at a single academic center with a relatively small sample size (*n* = 12) in the vaccinated group, reflecting the early adoption phase of the vaccine in Japan. Second, we did not longitudinally evaluate the decay rate of maternally transferred antibodies during the first six months of life, nor did we measure neutralizing antibody titers, which may correlate more directly with clinical protection than binding IgG titers. Future large-scale, multicenter longitudinal studies are required to confirm the duration of protection and the real-world effectiveness of maternal vaccines in the Japanese population.

## 5. Conclusions

In conclusion, maternal immunization with Abrysvo significantly enhances RSV pre-F IgG titers in Japanese neonates compared with natural maternal immunity. Given the remarkably lower antibody levels observed in infants hospitalized with RSV infection, expanding maternal vaccination coverage is an important public health strategy to reduce the burden of severe pediatric RSV infections in the post-pandemic era.

## Figures and Tables

**Table 1:** Baseline characteristics of neonates at birth.

Parameter	Unvaccinated (*n* = 40)	Vaccinated (*n* = 12)	*p*-Value
**Gestational age (weeks)**, median (IQR)	38 (37–39)	38 (37–39)	0.58
**Male sex**, *n* (%)	26 (65.0%)	5 (41.7%)	0.19
**Mode of delivery (Vaginal)**, *n* (%)	22 (55.0%)	3 (25.0%)	0.10
**Birth weight (g)**, median (IQR)	2844 (2550–3120)	2916 (2680–3240)	0.49
**Birth weight category**, *n* (%)			
Low birth weight (1500–2499 g)	11 (27.5%)	4 (33.3%)	0.71
Normal birth weight (≥2500 g)	29 (72.5%)	8 (66.7%)	0.71
**5 min Apgar score**, median (IQR)	9 (9–10)	9 (9–10)	0.20
**Presence of older siblings**, *n* (%)	13 (32.5%)	7 (58.3%)	0.11
**Palivizumab/Nirsevimab at birth**, *n* (%)	14 (35.0%)	1 (8.3%)	0.10

**Table 2:** Demographic and clinical characteristics of the hospitalized RSV infant cohort (*n* = 18).

Parameter	Hospitalized RSV Cohort (*n* = 18)
**Age at admission (months)**, median (IQR)	2 (1–4)
**Male sex**, *n* (%)	13 (72.2%)
**Vaginal delivery**, *n* (%)	18 (100.0%)
**Presence of older siblings**, *n* (%)	15 (83.3%)
**Severe RSV-associated LRTI** ^a^, *n* (%)	11 (61.1%)
**Maternal Abrysvo vaccination**, *n* (%)	0 (0.0%)
**Infant immunoprophylaxis** ^b^, *n* (%)	0 (0.0%)

^a^ Defined as requiring hospitalization for acute lower respiratory tract infection (LRTI) presenting with significant respiratory distress (tachypnea, chest retractions, or SpO_2_ < 92% on room air) necessitating oxygen therapy. ^b^ History of palivizumab or nirsevimab administration. Note that this infected clinical cohort is completely separate and independent from the healthy neonatal cohorts evaluated at birth in Table 1.

**Table 3:** Serum RSV pre-F IgG geometric mean titers (GMTs) across cohorts and clinical sub-characteristics.

Cohorts and Characteristics	*n*	RSV pre-F IgG GMT (95% CI)	*p*-Value
Main Cohort Comparison			<0.001
Neonates of Vaccinated Mothers (Cord Blood)	12	4.74 (4.30–5.18)	
Neonates of Unvaccinated Mothers (Cord Blood)	40	3.80 (3.50–4.14)	
Hospitalized RSV Infants (Peripheral Blood)	18	1.08 (0.65–2.15)	
By Gestational Age (All Neonates at Birth)			0.42
36–37 weeks	14	3.91 (3.45–4.32)	
38–39 weeks	33	4.12 (3.80–4.45)	
≥40 weeks	5	3.85 (3.20–4.21)	
By Birth Weight (All Neonates at Birth)			0.65
1500–2499 g (Low Birth Weight)	15	3.95 (3.51–4.38)	
≥2500 g (Normal Birth Weight)	37	4.08 (3.78–4.39)	
By Clinical Severity (Hospitalized RSV Cohort)			0.88
Mild/Moderate LRTI	7	1.12 (0.58–2.22)	
Severe LRTI	11	1.05 (0.61–2.11)	

## Data Availability

The raw data supporting the findings of this study are available from the corresponding author upon reasonable request.

## Abbreviations

The following abbreviations are used in this manuscript:

ALRTI	Acute Lower Respiratory Tract Infection
AMED	Japan Agency for Medical Research and Development
ANOVA	Analysis of Variance
CI	Confidence Interval
COVID-19	Coronavirus Disease 2019
ELISA	Enzyme-Linked Immunosorbent Assay
FcRn	Neonatal Fc Receptor
GMT	Geometric Mean Titer
IgG	Immunoglobulin G
IQR	Interquartile Range
NPI	Non-Pharmaceutical Intervention
RSV	Respiratory Syncytial Virus
